# Effects of Electrical Stimulation of the Cell: Wound Healing, Cell Proliferation, Apoptosis, and Signal Transduction

**DOI:** 10.3390/medsci11010011

**Published:** 2023-01-17

**Authors:** Kazuo Katoh

**Affiliations:** Laboratory of Human Anatomy and Cell Biology, Faculty of Health Sciences, Tsukuba University of Technology, Tsukuba 305-8521, Japan; katoichi@k.tsukuba-tech.ac.jp

**Keywords:** signal transduction, electrical stimulation, cultured cells, cytoskeleton, cell adhesion

## Abstract

Electrical stimulation of the cell can have a number of different effects depending on the type of cell being stimulated. In general, electrical stimulation can cause the cell to become more active, increase its metabolism, and change its gene expression. For example, if the electrical stimulation is of low intensity and short duration, it may simply cause the cell to depolarize. However, if the electrical stimulation is of high intensity or long duration, it may cause the cell to become hyperpolarized. The electrical stimulation of cells is a process by which an electrical current is applied to cells in order to change their function or behavior. This process can be used to treat various medical conditions and has been shown to be effective in a number of studies. In this perspective, the effects of electrical stimulation on the cell are summarized.

## 1. Introduction

In the field of acupuncture and moxibustion, electrical stimulation of the skin and muscles is known to locally increase blood flow and metabolism and maintain the body in a sustained healthy state [1]. However, little is known about the changes in cellular morphology and the localization of specific signal transduction proteins associated with electrical stimulation. From this perspective, the author will summarize the effect of electrical stimulation on proteins related to signal transduction.

The author’s recent work demonstrated that when cultured fibroblasts were periodically electrically stimulated (50 V, 60 times/min cyclically), the stress fibers in the cells became thicker, and the cells contracted [2]. When the cells were subjected to periodic electric stimulation overnight, the stress fibers became thicker. In addition, after approximately two hours, the stress fibers and focal adhesions in the cells had enlarged, the former exhibiting a growth in thickness, while the cells had a contracted appearance. After the electrical stimulation, phosphotyrosine-containing proteins accumulate in the focal adhesions of the cells. The active form of focal adhesion kinase (FAK) and activated form of c-Src, which are localized signal transduction proteins, had also accumulated in the focal adhesions. This observation indicated that proteins related to certain kinds of signal transduction were affected by the electrical stimulation [2,3,4]. From this perspective, the author will summarize the effect of electrical stimulation on signal transduction proteins.

## 2. Electrical Stimulation of Cells

### 2.1. Cell Proliferation and Apoptosis

Exogenous electrical stimulation is frequently used to manipulate cells to induce changes in various cellular processes, such as apoptosis and cell proliferation. This section first outlines the mechanisms involved in exogenous electrical stimulation during the inflammation, proliferation, and remodeling stages of the wound healing process [5,6].

#### 2.1.1. Inflammatory Phase in the Healing Process of Wounds

The inflammatory phase starts as soon as wound formation and involves the coagulation cascade, inflammatory pathways, and immune system activation. Electrical stimulation promotes vasodilation and increases cellular vascular permeability, allowing more white blood cells, platelets, and other cells to collect at the wound site. As an example of cell migration by electrical stimulation, Hoare et al. [7] reported that macrophages migrate to the anode with very low stimulation (5 mV/mm) and that the rate of directional migration is proportional to electric field strength, resulting in increased phagocytosis of oxalate microspheres, C-type albicans, and apoptotic neutrophils. These results suggest that the electric field, at the molecular level, activated ERK and P13K pathways [8,9], which then increased intracellular Ca^2+^ influx, including TRPV2-like Ca^2+^ influx in macrophages, which would enhance bacterial phagocytosis efficiency [10].

In addition to its impact on the eukaryotic cells, milliampere (0.2–140 mA) direct current (DC) inhibits the growth of *E. coli* [11], while alternating current (AC) of similar magnitude (15–30 mA) produces only negligible effects. Another study also reported that lower intensity (0.4–400 μA) DC inhibits the growth of *Staphylococcus aureus* [12].

#### 2.1.2. Proliferative Phase in the Wound Healing Process

During the proliferative phase of wound healing, re-epithelialization, fibrogenesis, and angiogenesis occur. Electrical stimulation has the effect of stimulating keratinocyte proliferation and differentiation, increasing keratin deposition and the keratinocyte migration rate, a prerequisite for re-epithelialization [13]. While reducing the inflammatory cytokines IL-6 and IL-8, electrical stimulation of keratinocytes also activates the ERK1/2 and p38 MAP kinase pathways [14]. Electrical stimulation also influences the orientation and directed migration of cultured corneal epithelial cells [15], migration of *Dictyostellium discoideum* [16], and re-epithelialization of murine corneal wounds [17]. The decrease in inflammatory cytokine expression suggests a smooth transition from the inflammatory to the proliferative phase, and thus electrical stimulation may promote wound healing.

Enhanced granulation tissue ingrowth into the wound center was shown in an in vivo study of human wounds subjected to monophasic and biphasic electrical stimulation at a field strength of 100 mV/mm for 30 min each day for 16 days. Immunohistochemical analysis revealed a doubling of epidermal thickness on day 16, significantly greater cytokeratin-10 staining, and almost three times higher cytokeratin-10 mRNA expression relative to controls [18]. Furthermore, in the corresponding ex vivo wound model, it was also observed that electrical stimulation significantly increased the expression of the following proteins: (1) proliferating cell nuclear antigen (PCNA), a DNA clamp that acts as a processivity factor for DNA polymerase delta; (2) human double minute 2 (HDM2), in which HDM2 forms a complex with p53, regulating the tumor suppressor functions of p53; and (3) SIVA1, which is an apoptosis-inducing factor. These proteins, which are all known to be involved in cell cycle regulation and DNA damage repair, were found to display different responses to the electrical stimulus. Thus, PCNA was seen to increase, while HDM2 decreased, with SIVA1 displaying no significant change [19].

It has also been reported that in fibroblasts, electrical stimulation leads to more collagen deposition and faster migration [14,20,21,22]. FGF-1 and FGF-2 secretion by fibroblasts is significantly upregulated by electrical stimulation, readjusting the balance of cell migration, proliferation, and differentiation [23]. Fibrogenesis is important for the granulation of tissue, and fibroblasts must be allowed to move to cover the wound. The directional migration of fibroblasts for this purpose was enhanced by electrical stimulation, with the localized clustering of integrin α2β1 and lamellipodia formation more prone to occur toward the cathode side of the electric field [24].

In addition, electrical stimulation promotes angiogenesis, which allows endothelial cells to migrate and increases blood supply to the wound. Previous studies using fibroblasts and human umbilical vein endothelial cells (HUVECs) have shown that electrical stimulation activates the NOS pathway and upregulates FGF2, which in turn activates the mitogen-activated protein kinase (MAPK)/ERK pathway cascade, and further promotes VEGF expression [25]. Fibroblasts were also found to increase FGF-1 and FGF-2 secretion after electrical stimulation; since FGF-1, FGF-2, and VEGF are angiogenic factors and need to be present for efficient wound healing, these increased expressions indicate the considerable therapeutic potential of electrical stimulation in wound healing. In studies on HUVECs and human mammary epithelial cells (HMECs), electrical stimulation increased the rate of cathodic migration of both kinds of cell, increased production of the C-X-C chemokine receptor type 4 (CXCR4) and type 2 (CXCR2), which are important for endothelial cell migration, and also caused the mitotic cleavage plane of both kinds of cell to be perpendicular to the field vector in contrast to the control’s random orientation [26,27].

#### 2.1.3. Remodeling Phase in the Wound Healing Process

Even at the end of the wound healing process, electrical stimulation accelerates the remodeling process by increasing the contractility of myofibroblast and the replacement of type-III collagen by type-I collagen through collagen fiber reorganization, enhancing maturity by increasing tensile strength.

Rouabhia et al. [28] used a collagen gel assay to demonstrate that both field intensity and exposure time were beneficial in causing fibroblasts seeded in collagen gels to contract more when electrically stimulated than those from control experiments. Studies using pulsed electrical stimulation also showed that electrically stimulated activated fibroblasts expressed significantly higher levels of α-SMA (smooth muscle actin) mRNA in qRT-PCR experiments [29]. Human skin fibroblasts receiving electrical stimulation exhibited increased tension and expressed higher levels of α-SMA and TGF-β1 in a collagen gel contraction assay. Thus, electrical stimulation may enhance the contractile capacity of fibroblasts by promoting trans-differentiation into myofibroblasts.

#### 2.1.4. Other Aspects of Wound Healing Facilitated by Electrical Stimulation

Electrical stimulation 14 days post-injury upregulated the expression of substance P, a neurotransmitter, and Protein Gene Product 9.5, a pan-neuronal marker, by 60 times and 30 times, respectively, in comparison to uninjured skin, indicating effective reinnervation [30]. In the same study, electrical stimulation upregulated class III-tubulin (TUBB3) and its upstream molecule, factor-inducible gene 4, and increased glycoprotein 100, a melanocyte system-specific antigen that indicates the existence of TUBB3+ melanocytes and the formation of melanin [31]. Thus, electrical stimulation promotes wound healing, reinnervation, and repigmentation of the wounded skin region.

Even though lymphatic drainage is not an essential factor in wound healing, lymphedema is linked to persistent wounds and inhibits wound healing [32]. In addition, via the FAK pathway, electrical stimulation has been demonstrated to promote the proliferation and migration of lymphatic endothelial cells [33,34].

### 2.2. Cell Proliferation and Apoptosis

While there are many reports that electrical stimulation of cells promotes apoptosis, there are also reports that it suppresses apoptosis and others that it has no effect at all. Since there are differences in the methods used in the studies, this subsection will give examples of studies showing these different results [35].

#### 2.2.1. Studies Showing Electrical Stimulation Promotes Apoptosis

Apoptosis is believed to be induced by the formation of pores in cell membranes by electrical stimulation. Specifically, the application of high-voltage electrical potential (HVEP) reversibly forms pores in the cell membrane, leading to apoptosis through the loss of cell membrane potential and disrupting the homeostasis of ions and other molecules [36]. Other studies have shown that low-voltage electrical pulses (LVEP) induce apoptosis by inducing activation of caspase-8 and caspase-9 and subsequent activation of caspase-3 [37,38]. Altered transport of extracellular molecules like Ca^2+^ involved in caspase activation has been suggested to trigger apoptosis in B16 cells, a melanoma cell line that expresses voltage-gated Ca^2+^ channels [39].

Alteration of the expression of the proteins p53 and Bcl-2 is another biological pathway by which electrical stimulation induces cell apoptosis. In studies targeting the HepG2 hepatocellular carcinoma cell line, induction of apoptosis by capacitive and resistive electric transfer (CRET) resulted in upregulation of p53, which was translocated into the nucleus in response to DNA damage at the end of a 24 h treatment period. The anti-apoptotic factor Bcl-2 was subsequently shown to be downregulated as a result [40].

#### 2.2.2. Studies Showing Electrical Stimulation Inhibits or Does Not Affect Apoptosis

**Figure 1:**
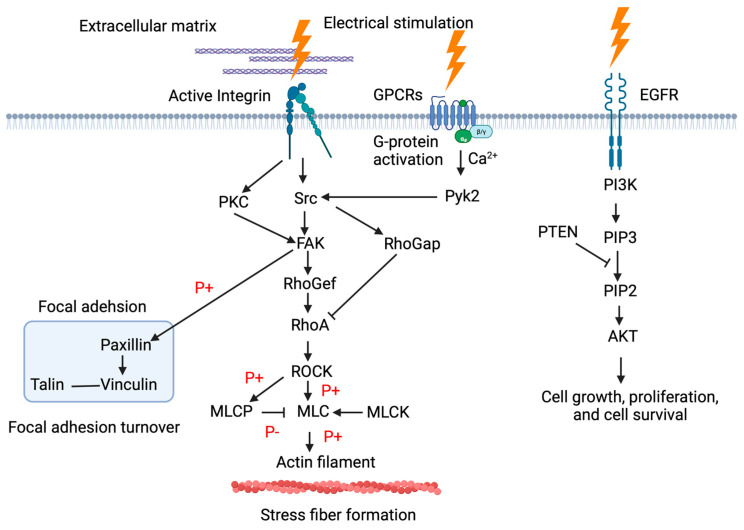
Schematic illustration of signal transduction within the cell after electrical stimulation.

Studies involving neuronal tissue, which is excitable, stimulated electrically, and voltage-gated ion channels, have reported that phosphoinositide 3-kinase-protein kinase B (PI3K-Akt) signaling pathway activation plays a role in preventing apoptosis [41]. Furthermore, an in vivo study using constant-current square wave electrical pulses to stimulate the rat cerebral cortex showed that, in comparison to controls, phosphorylated Akt was upregulated in both the cortex and striatum [41]. These results indicate that electrical stimulation causes activation of the PI3K-Akt pathway and suppresses cell apoptosis (Figure 1).

Electrical stimulation of G-protein coupled receptors (GPCRs) would be a candidate for the influx of Ca^2+^ into the cell, causing the membrane potential to become more depolarized and thus allowing positive ions, such as Ca^2+^, to enter the cell. Alternatively, electrically activated integrins affect the activation of c-Src, resulting in FAK in downstream signaling events that regulate the formation of focal adhesion and stress fiber organization [2].

In contrast, the EGFR signaling pathway is crucial in the regulation of cellular growth, proliferation, and differentiation. It involves the activation of various proteins and signaling molecules, such as PI3K and Akt [42,43].

In addition to activating the PI3K-Akt pathway, constant-current square wave electrical pulses activate the MAPK pathway in studies examining the protective effects of constant-current square wave electrical pulses on gastric mucosal tissue after gastric ischemia-reperfusion injury (GI/RI). Gastric mucosal cells’ (GMCs’) apoptotic rate following GI/RI injury has also been reported to be reduced when the rat paraventricular nucleus (PVN) is electrically stimulated with constant-current square wave electrical pulses [44]. Furthermore, the phosphor-c-Jun terminal kinase (p-JNK) and p38 mitogen-activated protein kinase (p38 MAPK) pathways were suppressed after PVN electrical stimulation.

However, several studies have shown that electrical stimulation does not affect cell apoptosis [19,45,46]. For example, keratinocytes [19], Schwann cells [47], and olfactory envelope cells [48], as well as vascular smooth muscle cells [45], were exposed to high-voltage DC (100 mV/mm) at 300–400 mV/mm, but no effect on apoptosis was observed in either case.

### 2.3. Effects of Stimulation on the Proliferation of Cells

Many studies have shown that electrical stimulation affects cell proliferation. In this subsection, the effects of electrical stimulation on cell proliferation will be described.

#### 2.3.1. Electrical Stimulation Promotes Cell Proliferation

“Increased proliferation rate” and “increased secretion of growth factors from electrically stimulated cells” have been linked in studies [47,48]. Neurotrophic factors, such as BDNF (brain-derived neurotrophic factor), GDNF (Glial cell-line derived neurotrophic factor), and NGF (nerve growth factor), are known to improve the survival of neurons. For example, the secretion of BDNF, GDNF [49], NGF [50], neuronal cell adhesion molecule (N-CAM) [51], and VEGF [15], was raised when DC (100 mV/mm) was applied through conductive composites whereas the secretion of neurite outgrowth inhibitor (NOGO-A) in olfactory envelope cells was decreased [52]. Meanwhile, an increase in BDNF and NGF levels has been reported in bone marrow MSCs [53]. Studies on olfactory bulb neural progenitor cells using biphasic electrical stimulation (B electrical stimulation) (25 mV/mm) have also discovered that elevated cell proliferation is accompanied by elevated BDNF release [52]. It is believed that these growth factors are upregulated to initiate an increase in cell proliferation.

Constant-current square wave electrical pulses also induce the ERK 1/2 pathway to be activated and the JNK and p38 MAPK pathways to be suppressed. These are due to the increased phosphorylated ERK activity. In addition, since it has been reported that inhibition of the ERK 1/2 pathway, even in the presence of electrical stimulation of PVN, decreases the proliferation rate of intestinal mucosal cells after I/R injury [54], it can be assumed that ERK 1/2 pathway activation is essential to increase cell proliferation.

As described above, among the types of applied electrical stimulus, there are several reported cases in which direct current suppresses cell proliferation.

So far, examples have been presented of electrical stimulation promoting and inhibiting cell proliferation, but there are also studies showing that electrical stimulation has no effect on cell proliferation [39,41,55].

#### 2.3.2. Electrical Stimulation Inhibits Cell Proliferation

It has also been shown in some studies that the application of DC alone inhibits the proliferation of certain types of cells: in vivo studies in rabbits in which vascular smooth muscle was stimulated with 300–400 mV/mm for 30 min each day over 1, 2, or 4 weeks resulted in decreased cell proliferation through increased expression of phosphatase and tensin homolog (PTEN)/p27, resulting in a decreased cell.

The PTEN gene residing on chromosome 10 [56] regulates cyclin-dependent kinase inhibitor 1B (p27kip1), which inhibits proliferation by preventing cells from advancing to the G1 phase [57]. Therefore, PTEN upregulation induced by electrical stimulation may prevent cell cycle progression through the effect of p27kip1. In addition, the application of DC to human mesenchymal stem cells (MSCs) for 10 min each day for seven days at 100 mV/cm inhibited the proliferation of MSCs and started differentiation into neuron-like cells [46].

At lower electric field strengths, collagen I upregulated by electrical was comparable with controls. However, at a stronger electric field (150 mV/mm) and higher frequency (60 Hz), degenerative wave (DW) maximally downregulated type 1 collagen in keloid fibroblasts, although DW electrical stimulation showed lower cytotoxic effects on normal fibroblasts than AC or DC [58]. Increasing the DC amplitude upregulated type 1 collagen and PAI-1 gene transcription in normal and keloid fibroblasts [59]. This demonstrates highly differential effects of specific types of electrical stimulation on human fibroblast collagen expression and cytotoxicity and identifies DW as a promising, novel therapeutic strategy for suppressing excessive collagen I formation in keloid disease. In addition, DW can enhance cutaneous wound healing in vivo [29].

## 3. Molecular Mechanisms in Wound Healing Affected by Electrical Stimulation

Studies have reported that electrical stimulation affects wound healing. In this section, the molecular mechanisms by which electrical stimulation affects wound healing will be described.

### 3.1. Molecular Mechanisms in Wound Healing

Wound healing is essential for maintaining the integrity of multicellular organisms. It has been proposed that the disruption of the epithelial layer of a cell instantly generates an endogenous electric field, which is important for wound healing. However, the identity of the signaling pathways that guide both cell migration and wound healing by electric fields is not fully understood at the genetic level.

In wound healing, signal transduction affected by electrical stimulation is a physiological process whereby electrical stimulation triggers the potential of cells and second messenger pathways, leading to cellular responses. This event is essential in the proper formation of connective tissue and re-epithelialization in wound repair, enabling structural and functional restoration [42,43]. Ca^2+^ influx, one of the starting points of signal transduction, is a crucial factor in the process of wound healing. Increased intracellular Ca^2+^ concentration caused by electrical stimulation plays a role in stimulating cells during wound healing processes [60].

Studies in which electric fields of intensity equal to that of endogenous electric fields were applied have shown that electric field stimulation induces activation of signal transduction proteins, such as c-Src and inositol phospholipid signaling, and polarization in the direction of cell migration; that genetic disruption of phosphatidylinositol-3-OH kinase-gamma (PI(3)Kγ) reduces the electric field-induced signal and arrests the directed movement of the epithelium in response to electrical signals; and that deletion of PTEN, a tumor suppressor promotes signaling and the electric field response [42,43]. In other words, it is suggested that PI(3)Kg and PTEN regulate electromotility.

**Figure 2:**
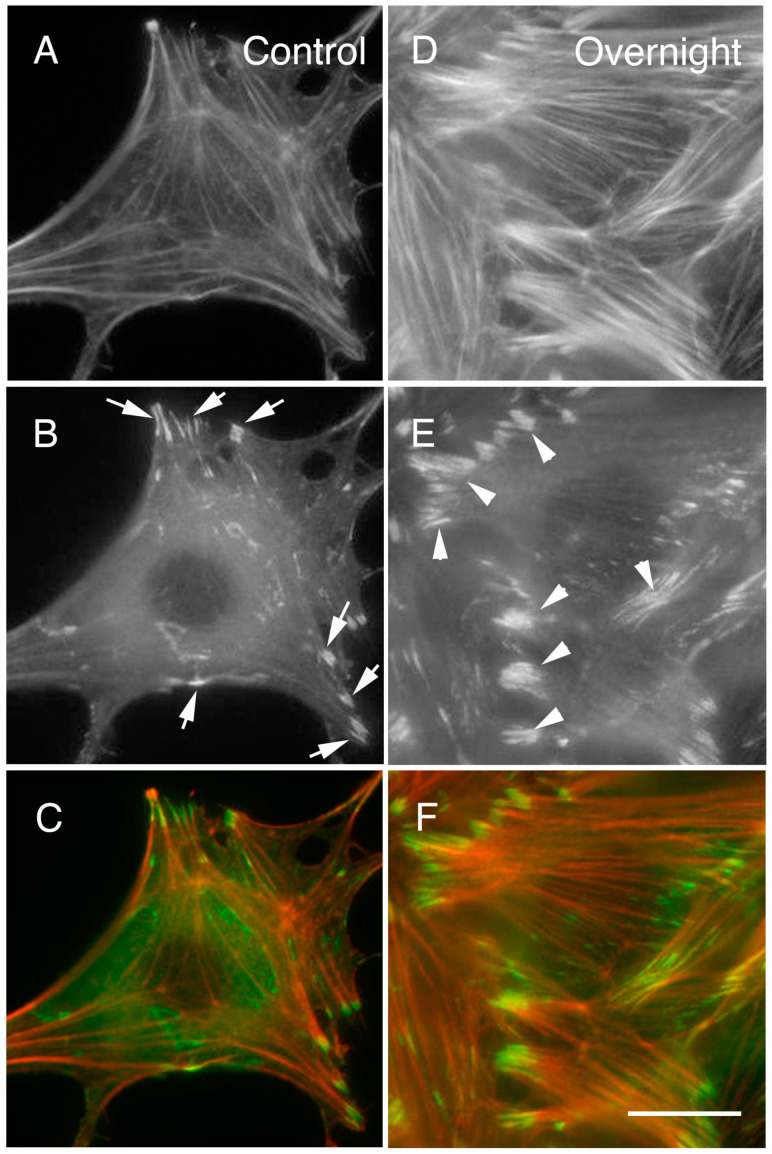
More and larger focal adhesions and stress fibers were observed after periodic electrical stimulation. An increase in the number and size of focal adhesions and stress fibers was observed after periodic electrical stimulation. After 20 h of such stimulation, both the stress fibers and focal adhesions had thickened and enlarged (**D**–**F**) compared with those in the control (no electrical stimulation) (**A**–**C**). (**A**–**C**): Control (no electrical stimulation); (**A**): Rhodamine phalloidin; (**B**): GFP-paxillin; (**C**): Merge (**D**–**F**): 20 h of electrical stimulation; (**D**): Rhodamine phalloidin; (**E**): GFP-paxillin; (**F**): Merge. The arrows in (**B**) indicate typical focal adhesion before electrical stimulation (control); the arrowheads in E indicate enlarged focal adhesions after electrical stimulation for 20 h. Bars: 10 μm (all figures are of the same magnification). See also Katoh (2022) [2].

Exposure of keratinocytes and neutrophils to an exogenous electric field in a serum-free medium also induced rapid and sustained phosphorylation of extracellular signal-regulated kinase (ERK), p38 mitogen-activated kinase (MAPK), and c-Src, as well as the phosphorylation of Akt (S473) in electrically migrating keratinocytes [13,61]. As a result, tyrosine phosphorylated c-Src polarized in the direction of migration. Furthermore, the phosphorylation of Janus kinase 1, JAK1, was unchanged, suggesting that the current activates only a defined signaling pathway [42,60]. The study demonstrated that cultured fibroblasts stimulated electrically have improved cytoskeletal structure. Molecular analyses showed that exogenous electrical stimulation of fibroblasts (50 V, 60 times/min cyclically for more than two hours) increased tyrosine phosphorylation of the Src family and focal adhesion kinase (FAK) as well as the number and size of focal adhesions and stress fibers [2] (Figure 2).

The relevant signal transduction processing is schematically illustrated in Figure 2.

The mechanism underlying the fibroblast response to electrical stimulation is believed to involve the transmission of information to the intracellular signaling system through signaling the molecule at the cell adhesion sites as FAK, and together with structural proteins including paxillin, talin, and vinculin [2].

### 3.2. Signal Transduction Mechanisms in Wound Healing

As mentioned above, at the molecular level, during the inflammatory phase, the initial phase of wound healing, electrical stimulation is believed to activate ERK and P13K pathways. This increases Ca^2+^ influx into the cell, such as TRPV2-like Ca^2+^ influx in macrophages, thereby increasing bacterial phagocytic efficiency [62,63]. Additionally, electrical stimulation of keratinocytes during the proliferative phase after the inflammatory phase activates the ERK1/2 and p38 MAP kinase pathways, which are linked to a reduction in the inflammatory cytokines IL-6 and IL-8 [14]. This is believed to inhibit inflammation and promote an efficient transition to the proliferative phase of the cell.

Studies with fibroblasts and HUVECs have shown that electrical stimulation activates the NOS pathway and upregulates EGF2, resulting in cascade activation of the MAPK/ERK pathway, which promotes VEGF expression. External DC electric fields can cause substantial actin cytoskeleton reorganization [9]. Therefore, it is believed that DC electric fields induce membrane redistribution, which in turn triggers signaling pathways by binding many growth factors such as EGF, FGF, and TGF-β1 to their corresponding receptors, resulting in local changes in actin dynamics. Another important receptor associated with EF is the epidermal growth factor receptor (EGFR) [64]. EFs of physiological intensity activate multiple signaling pathways, including EGFR, ERK, Src, PI3K, and MAPK signaling during exposure for an extended period. Other studies show that electrical stimulation can activate plasma membrane receptors coupled to phospholipase C (PLC), thereby activating the PLC signaling cascade and releasing Ca^2+^ from the endoplasmic reticulum.

The chemokine receptors CXCR4 and CXCR2, which are important for endothelial cell migration, are produced more abundantly in response to electrical stimulation [26]. Fibroblasts activated by electrical stimulation have also been found to express noticeably increased levels of α-SMA mRNA in qRT-PCR. Electrical stimulation also considerably increases the expression of substance P (a neurotransmitter) and Protein Gene Product 9.5 (a pan-neuronal marker) by 60 times and 30 times, respectively, indicating that such stimulation causes post-wound cellular reinnervation.

## 4. Short Summary

In promoting the healing of wounds by electrical stimulation, questions arise about the changes in cellular morphology and the localization of specific signal transduction proteins associated with such stimulation. In this paper, the author, having demonstrated that proteins related to certain kinds of signal transduction are affected by electrical stimulation, considers the findings of other researchers on the effects of such stimulation on electrically non-excitable and excitable cells.

According to studies, an electric field activates ERK and P13K pathways at the molecular level, increasing intracellular Ca^2+^ influx in macrophages and improving the efficiency of bacterial phagocytosis. The growth of *Staphylococcus aureus* is inhibited, and re-epithelialization, fibrogenesis, and angiogenesis occur. Monophasic and biphasic electrical stimulation promotes granulation tissue ingrowth into the center of a wound, but activation of keratinocytes also stimulates the ERK1/2 and p38 MAP kinase pathways and lowers the inflammatory cytokines IL-6 and IL-8.

Electrical stimulation activates the NOS pathway and upregulates FGF2, which leads to the activation of the mitogen-activated protein kinase (MAPK)/ERK pathway cascade, and further promotes VEGF expression. Fibroblasts have been found to increase FGF-1 and FGF-2 secretion after electrical stimulation. The chemokine receptors CXCR4 and CXCR2, which are important for endothelial cell migration, are produced more abundantly as a result of such stimulation. Pulsed electrical stimulation of activated fibroblasts produces significantly higher levels of a-SMA mRNA, while stimulation of human skin fibroblasts produces higher levels of α-SMA and TGF-β1. Electrical stimulation upregulates the expression of substance P and Protein Gene Product 9.5, indicating successful reinnervation. It also upregulates class III-tubulin and its upstream molecule, factor-inducible gene 4, and increases glycoprotein 100, indicating the formation of melanin.

With respect to apoptosis, wherein electrical stimulation is believed to form pores in cell membranes, low-voltage electrical pulses induce apoptosis by activating caspase-8, caspase-9, and caspase-3. This suggests that considering B16 cells, a melanoma cell line that expresses voltage-gated Ca^2+^ channels, altered transport of extracellular substances, including Ca^2+^, may be involved in caspase activation. Furthermore, hepG2 hepatocellular carcinoma cell line-specific capacitive and resistive electric transfer to induce apoptosis upregulates p53, which is translocated into the nucleus in response to DNA damage, downregulating the anti-apoptotic factor Bcl-2. Meanwhile, electrical stimulation of neuronal tissue, which is excitable, and voltage-gated ion channels, activates the phosphoinositide 3-kinase-protein kinase B (PI3K-Akt) signaling pathway, which is crucial in inhibiting apoptosis. After a gastric ischemia-reperfusion injury, constant-current square wave pulses activate not only the PI3K-Akt pathway but also the MAPK pathway of the gastric mucosal tissue. However, some studies have shown that electrical stimulation does not affect cell apoptosis.

In wound healing, applying electric fields of intensity equal to that of endogenous electric fields activates signal transduction proteins, including c-Src, and inositol phospholipid signaling. At the molecular level, during the inflammatory phase of healing, electrical stimulation is believed to activate ERK and P13K pathways, increasing Ca^2+^ influx into the cell, such as TRPV2-like Ca^2+^ influx in macrophages, thereby increasing bacterial phagocytic efficiency. Electrical stimulation involving fibroblasts and HUVECs activates the NOS pathway and upregulates EGF2, resulting in cascade activation of the MAPK/ERK pathway, which promotes VEGF expression. Electrical stimulation can also activate plasma membrane receptors coupled to phospholipase C, thereby activating the PLC signaling cascade and releasing Ca^2+^ from the endoplasmic reticulum. The chemokine receptors CXCR4 and CXCR2, which are important for endothelial cell migration, are produced more abundantly in response to such stimulation.

A list of signal transduction proteins influenced by electrical stimulation appearing in this perspective is shown in Table 1.

## 5. Conclusions

In conclusion, the effects of electrical stimulation applied to a cell are varied and dependent on a number of factors. These include the type of cell, the intensity and duration of the stimulation, and the specific location of the cell being stimulated.

Electrical stimulation has been demonstrated to activate signal transduction pathways within the cell, such as MAPK pathways which transmit extracellular signals into the nucleus, and integrin-associated signal transduction proteins, such as Src family tyrosine kinases, FAK and Rho A, which are involved in cell proliferation and cell adhesion. These signal transduction proteins can be stimulated electrically, resulting in a cascade of intracellular events that can ultimately alter cellular physiology. This activation is mediated by the influx of ions through specialized channels, such as voltage-gated ion channels or mechanosensitive channels, allowing for the flow of electrical charge across the cell membrane into the inside of the cells. Electrical stimulation can have both positive and negative effects on the cell, depending on the circumstances. For example, if the electrical stimulation is of low intensity and short duration, it may simply cause the cell to depolarize. However, if the electrical stimulation is of high intensity or long duration, it may cause the cell to become hyperpolarized.

## Figures and Tables

**Table 1:** List of signal transduction proteins influenced by electrical stimulation appearing in this manuscript.

Factors	Protein Names
Ca^2+^ Influx	Ca^2+^ Channels [1,2]
Signal transduction proteins to the nucleus	EGFR [3] ERK [4,5,6,7] PI3K [4,5,8,9] TRV2 [10] Atk [11] ERK1/2 [12] p38 MAP kinase [12,13] MAPK [14] JNK [13,15]
Cell adhesion and cytoskeletal proteins	Integrin *α*2*β*1 [16] FAK [17,18] Src [17,18] *α*-SMA [19] TUBB3 [20] cytokeratin-10 [21]
Neurotransmitter proteins	substance P [22] PGP (Protein Gene Product) 9.5 [22]
Apoptosis associated proteins	P53 [23] Bcl-2 [23] PCNA [24] HDM2 [24] SIVA1 [24]
Cytokines, Chemokines	IL6 [12] IL 8 [12] CXCR2 [25,26] CXCR4 [25,26]
Neurotrophic Factors	BDNF [29,30] GDNF [29] NGF [30,31] N-CAM [32] NOGO-A (neurite growth inhibitor) [33] PTEN (inhibit PI3kinase) [34] p27kip1 (cell cycle inhibitor) [35]

## Data Availability

The data presented in this study are available on request from the corresponding author.
